# Use of EDTA and CaCl_2_ Extraction Methods to Predict the Bioavailability of Heavy Metals in Soils Polluted with Microplastics

**DOI:** 10.3390/ma18040760

**Published:** 2025-02-09

**Authors:** Bhakti Jadhav, Agnieszka Medyńska-Juraszek

**Affiliations:** Institute of Soil Science, Plant Nutrition and Environmental Protection, Wroclaw University of Environmental and Life Sciences, 53 Grunwaldzka Str., 50-357 Wrocław, Poland; bhakti.jadhav@upwr.edu.pl

**Keywords:** soil bioavailability, metal mobility, heavy metals, microplastics, EDTA extraction, CaCl_2_ extraction, soil contamination, environmental pollution

## Abstract

Microplastic (MP) contamination in soil is an emerging environmental concern, influencing the mobility and bioavailability of heavy metals (HMs). This study investigates how different MP types (PP, PS, PVC, HDPE, LDPE, PES, and PET-Glitter) affect HM behavior in soil, focusing on sorption/desorption, and the extraction efficiency of Pb, Cu, Co, Ni, Cr, and Cd. Soil samples incubated with MPs showed significant pH increases, particularly with PES and HDPE at 0.8 and 0.6 pH units, respectively. The extraction experiments using 0.05 M EDTA and 0.01 M CaCl_2_ revealed that MPs altered metal bioavailability—with HDPE reducing Pb mobility by 15%—and increased Cd and Co mobility by 10–20%. The batch sorption tests confirmed higher Pb adsorption onto HDPE but decreased Cd and Co sorption compared to control soils without MP. These findings demonstrate that MPs act as additional sorption sites, modifying metal speciation and availability, which has critical implications for soil health, agricultural sustainability, and remediation strategies. However, results may vary based on soil type, MP aging, and environmental conditions, indicating the need for further long-term field studies. This research provides valuable insights into the complex interactions between MPs, heavy metals, and soil systems, contributing to a better understanding of pollution dynamics and risk assessment in contaminated environments.

## 1. Introduction

There is growing concern about microplastics entering the food chain, as the surface of chemically active microplastics (MPs) allows them to absorb toxic contaminants, e.g., heavy metals [1], modifying their mobility and bioavailability in soil and, thus, causing a risk of higher accumulation of metals in plants and micro-organisms. The combination of MPs and potentially toxic elements (PTEs), such as heavy metals (HMs), in soil represents a significant threat to food production and human health, as the mobility and bioavailability of PTEs may increase or decrease in the presence of different MP polymers but also depends on interactions between metals and MP surfaces. Studies related to microplastic interactions in soil and changes in heavy metal mobility and bioavailability are rare. In terms of predicting metal mobility in soil in the presence of new types of particles that are not a part of natural soil constituents but are prone to chemical changes on their surface, the mechanism of heavy metal sorption/desorption in soil may be the result of the surface properties of the microplastic itself as well as the interactions between MPs and the components of the soil sorption complex, such as organic matter and clay minerals. Previously, the interactions between metals and plastics were omitted. Recent studies have revealed that the adsorption of PTEs onto microplastics depends on many factors, including electrostatic forces, the pH of the medium, salinity, or the presence of organic matter [2], which seem to play crucial roles in controlling adsorption and desorption processes on microplastic surfaces. Microplastics (MPs) serve as sorption sites for heavy metals (HMs) in the environment due to their high surface area, hydrophobicity, and the presence of functional groups that facilitate contaminant binding. The sorption mechanisms include electrostatic interactions, complexation, ion exchange, and van der Waals forces. Aging processes, such as photodegradation, thermal degradation, biodegradation, and mechanical fragmentation, alter the surface properties of MPs, enhancing their capacity to adsorb HMs. For instance, aging increases surface roughness and introduces oxygen-containing functional groups, which can enhance HM sorption [3]. The adsorption capacity of MPs for PTEs is mainly affected by their properties (species, surface area, aging status, and functional groups), the properties of heavy metal ions (species and concentration), and environmental factors (pH and ionic strength) [4]. Subsequently, several studies indicate that different types of polymers, e.g., polyethylene terephthalate (PET), polyvinyl chloride (PVC), polypropylene (PP), or polystyrene (PS), can absorb various heavy metals; however, their affinity to bind to particular metals can be different [5], similar to interactions with (SOM) soil organic matter. Soil organic matter significantly influences HM binding to MPs. SOM contains functional groups like carboxyl, phenolic, and hydroxyl groups that have a high affinity for HMs, potentially competing with MPs for metal sorption. Additionally, dissolved organic matter (DOM), a component of SOM, can complex with HMs and increase their mobility, potentially reducing their direct adsorption onto MPs. Furthermore, SOM can form coatings on MPs, altering their charge and hydrophobicity, which may either enhance or reduce HM adsorption depending on environmental conditions [6]. The mobility of heavy metals in soil relates to their capacity to pass from the solid part of the soil to the soil solution, which determines the bioavailability and phytoavailability of the element in the soil. This mobility can be modified directly or indirectly by the presence of MP particles in the solid and aquatic phases of soil. It can also be directly modified by the properties of MPs, such as surface charge, surface functional groups, and surface roughness change during the MP aging process in soil [7]. Aging processes significantly affect the interaction between MPs and HMs. For example, UV irradiation can increase the adsorption capacity of MPs for HMs by altering their surface properties. Studies have shown that aged MPs exhibit higher adsorption capacities for metals like cadmium (Cd^2+^), copper (Cu^2+^), and zinc (Zn^2+^) compared to their pristine counterparts [8]. They can be indirectly modified by the wide impact of the physical (aggregation, bulk density, porosity, and water retention capacity), chemical (soil pH, organic matter, and nutrient cycling), and biological (microbial community and activity) properties of soil [9]. Some studies also suggest that the shape of microplastic particles (fragments, spheres, foams, films, and fibers) may also affect different soil properties and act differently as metal sorption sites. For example, MP fibers may increase water-holding capacity and soil porosity differently than thin fragments [10]. Microplastic surface properties can also be changed due to aging. Oxidation processes and weathering usually increase microplastic sorption capacity for inorganic and organic contaminants [11]. However, the interactions with aged microplastic surfaces might be distinct from interactions with pristine materials, and it is more probable that binding strength increases on aged MP surfaces; hence, the efficiency of extraction can be decreased. It has also been noticed that dissolved organic matter strongly enhances the mobility of microplastic particles, and fulvic acids present in soil enhance the sorption capacity of MP for metal ions [12]. Several techniques to evaluate the availability of HMs in soil have been described in single heavy metal extraction protocols. The extraction of heavy metals by unbuffered salt solutions like calcium chloride (CaCl_2_) is a simple way to evaluate the amount of metal fraction easily available to soil biota and plant forms. However, various studies have indicated that salt solutions may not accurately reflect plant-accessible metals compared to ethylenediaminetetraacetic acid (EDTA), which has shown greater predictability [13,14]. The extraction capacity of the metals was found to be of the order EDTA > Mehlich 3 > Mehlich 1 > (Diethylenetriaminepentaacetic acid) DTPA–TEA > NH_4_OAc > CaCl_2_ [15]. We assume that microplastic affects not only the bioavailability and mobility of heavy metals in soil but also the conditions and efficiency of the extraction of heavy metals commonly used in metal bioavailability analysis reagents. Microplastics may have a wide impact on soil structure, altering some of the physical characteristics of soil, such as porosity or water retention, thus affecting the distribution and accessibility of heavy metals and potentially impacting the strength of metal binding with soil constituents and metal speciation [15,16,17]. Microplastic particles can also serve as additional sorption sites, having high specific surface area and functional groups [18] that absorb metal ions under different strengths. The adsorption on the MP surface can reduce the availability of metals and chelation with, e.g., EDTA, thereby decreasing the effectiveness of the extraction process. According to studies, different microplastic types have different capacities for metal sorption. More hydrophobic polymers, like polyethylene (PE) or polypropylene (PP), bind metals less effectively than polar polymers, like polyethylene terephthalate (PET) or polyamides (PA); thus, polar microplastic may compete with extractants for binding metals. Turner and Holmes [19] found that the capacity for Pb (II) sorption by MPs is in the following order: polyvinyl chloride (PVC) > polypropylene (PP) > polyamide (PA) > polyethylene (PE) > polyoxymethylene (POM). Llorca et al. [20] also reported that polyethylene terephthalate and polystyrene (PS) had a higher affinity for pollutants than PE. Microplastic can also modify soil chemical characteristics such as soil pH, ionic strength, sorption capacity, or redox conditions [21], thus influencing metal speciation, solubility, and mobility in soil and the efficiency of metal extraction with chelating agents or unbuffered salts. Finally, there is doubt about whether EDTA or other reagents can be adsorbed onto microplastic, reduce the concentration of the solution, and thus reduce the efficiency of the metal extraction process from a soil sample. Specific studies examining the influence of microplastic on the extraction of heavy metals from soil are limited.

This study aims to analyze the problem of metal bioavailability in soil in the presence of microplastic contamination. Two extractants, commonly used in soil analysis, with different extraction strengths (0.05 M EDTA and 0.01 M CaCl_2_) were chosen for investigation. The extractability of lead (Pb), copper (Cu), cobalt (Co), nickel (Ni), chromium (Cr), and cadmium (Cd) from soil under the presence of seven different plastic polymers was tested. One of the goals of the study was to also investigate the changes in metal sorption/desorption conditions under the presence of microplastic particles in soil. The incubation and batch experiment using six metals and seven types of polymers was conducted to evaluate the possible changes in metal availability and extractability from soil in the presence of microplastic contamination. By studying how substances are absorbed at these boundaries, we aimed to uncover information about how microplastics, soil, and heavy metals interact. This research will help us better grasp the complexities of pollution. Through these studies, our main objective is to improve our understanding of the connections between microplastics, heavy metals, and soil elements. This awareness will offer guidance for creating better approaches to assess and address environmental issues.

## 2. Method and Materials

Soil for the batch experiment was collected from a buried soil profile to avoid additional contamination with microplastics. The soil had a texture of loamy sand (87% sand, 6% silt, and 7% clay). Microplastics were obtained by cutting, grinding, and sieving (2 mm mesh) polypropylene (PP), polystyrene (PS), polyvinyl chloride (PVC), high-density polyethylene (HDPE), low-density polyethylene (LDPE), polyester (PES), polyethylene terephthalate glitter (PET-Glitter) beads and flakes purchased from Sigma-Aldrich (St. Louis, MO, USA) and commercially available sources, e.g., art shops selling glitters and plastic flakes. The size of the microplastics ranged from 100 microns to 2 mm. All chemical reagents—EDTA and CaCl_2_—and Pb, Cu, Co, Ni, Cr, and Cd acetate salts at analytical grade were purchased from Chemlab (Piekoszów, Poland) and Sigma-Aldrich.

### 2.1. Incubation Experiment

**Figure 1:**
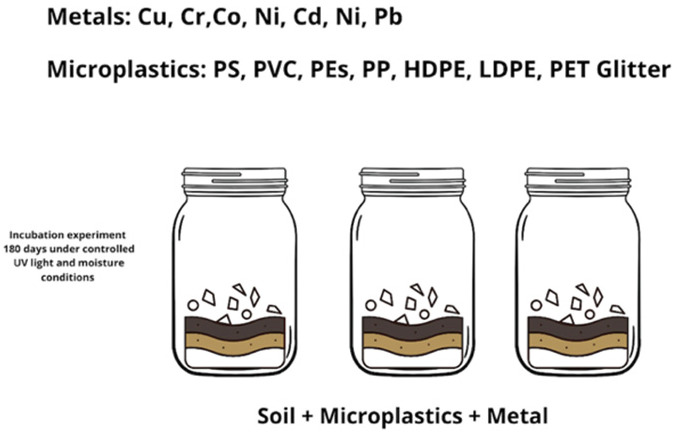
Incubation experiment for metal sorption in soils with different MPs.

Air-dry soil was sieved through a metal mesh to remove >2 mm particles. The initial soil composition, before metal addition, was free of detectable metallic elements. Spiking solutions with known concentrations of metals: chromium (Cr, 15 mg/L), copper (Cu, 10 mg/L), nickel (Ni, 15 mg/L), cobalt (Co, 15 mg/L), cadmium (Cd, 15 mg/L), lead (Pb, 50 mg/L) were obtained by dissolving the acetate salts (Chemland, Stargard, Poland) of metals in milli-Q water (MilliPore Merck, Burlington, MA, USA). To reach equilibrium, the spiking solutions were mixed for 24 h on a horizontal shaker. A total of 1 kg of soil was placed as a thin layer on a tray and sprayed with 25 mL of spiking solution. The procedure was repeated to obtain the proper amount of soil spiked with each metal for the incubation experiment. A total of 300 g of metal-spiked soil was placed in borosilicate glass media bottles (Shott, Mainz, Germany) mixed with 0.025 g (25 mg) of each microplastic; varying sizes were added, resulting in a 12,000:1 (soil: plastic) ratio by weight. The amount of microplastic added in the experiment was calculated based on the literature reviews describing the number of MP particles present in soil, and the soil-to-water ratio was 1:5, where 5 g of dry soil was mixed with 25 mL of extractant solution (0.05 M EDTA or 0.01 M CaCl_2_), as per standardized extraction protocols [15,22]. This ratio was used for both the metal bioavailability assessment and pH measurement, ensuring comparability between treatments. A total of 100 particles of glitter with a diameter of 1 × 1 mm were weighed on a laboratory scale at a precision of 0.0001 g (RadWag, Radom, Poland) to obtain the proper mass of the microplastic sample added to the soil. Incubation was performed for 6 months under controlled light and moisture conditions (Figure 1). Glass jars were kept in the dark to protect plastic and metal molecules from UV light. The soils were watered every other day using spray bottles for a period of up to 6 months. In total, 56 samples in two replicates were prepared with different combinations of heavy metals and microplastic content.

### 2.2. Batch Experiment

**Figure 2:**
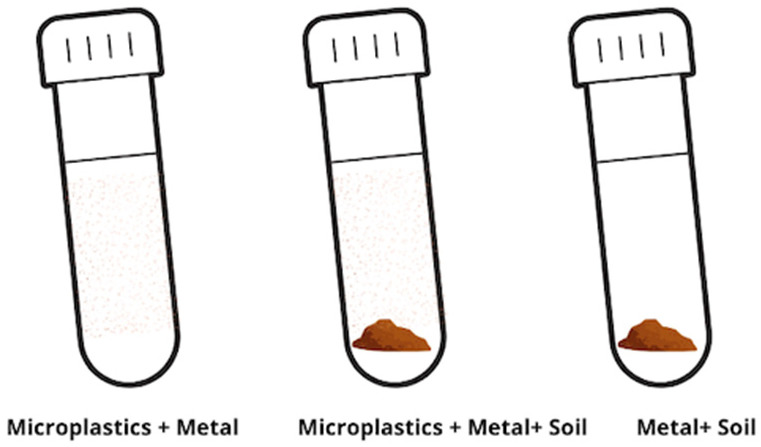
Batch experiment of microplastics and the soil sorption of heavy metals.

The removal of tested metals from the solution by three different media was performed to describe the efficiency and changes in metal adsorption in soil in the presence of microplastic (Figure 2). For this experiment, only one of the tested polymers—HDPE—was used, as this type of microplastic showed a significant impact on soil pH and metal mobility in our preliminary studies [23]. In the experiment, the sorption of Cu, Cd, Co, Cr, Ni, and Pb was tested on the following: pure HDPE microplastic (1), pure soil used previously in the incubation experiment (2), and a mix of soil and HDPE-MP (3). The same mass of sorbent—1.025 g—was used in all replicates. A stock solution of metals was prepared by diluting metal salts in milli-Q water (as described above). The concentration of metal was analyzed on an MP-AES 4200 Microwave Plasma Atomic Emission Spectroscope (Agilent Technologies, Santa Clara, CA, USA) before application to the sorbent. The concentration of metal in the solution is 30 ± 2.5 mg/L. A mixture of soil and microplastic was prepared by adding 0.025 g of HDPE (particle size < 2 mm) to 1 g of soil and thoroughly homogenizing the sample before further analysis, maintaining a soil-to-plastic ratio of 40:1. To ensure uniform mixing and facilitate metal sorption studies, 25 mL of each metal solution (Cd, Pb, Cu, Cr, Co, Ni) was added to this mixture, resulting in a soil-to-water ratio of 1:25. This ratio was selected based on previous studies evaluating metal sorption in microplastic-amended soils [15,22]. The standardized ratios ensure that microplastic interactions with soil and heavy metals are accurately assessed under controlled conditions and shaken in 50 mL Falcon tubes on a vertical shaker (Biosan, Riga, Latvia). A total of 5 mL of supernatant was collected after 24 h, 48 h, and 72 h for metal concentration analysis. According to previous research, the efficient increase in adsorption of metal(oid)s onto surfaces is frequently observed until equilibrium time, which is normally reached over 24 or 48 h for an aqueous solution [14].

The amount of metal adsorbed on different types of microplastics, microplastic added to the soil, and pure soil was calculated based on the difference in metal concentrations in an aqueous solution before and after the adsorption experiment according to Equation (1).

(1) $$\mathrm{qt}=\frac{\left(C_{0}-C_{t}\right)xV}{m}$$

where qt is the amount of metal adsorbed per unit weight of adsorbent (mg/g) at time t, *C*_0_ and *C_t_* are the concentrations of metal (mg/L) at an initial time and at 24 h, 48 h, and 72 h, respectively, *V* is the initial volume of metal solution (L), and *m* is the mass of adsorbent (g). Similar methods have been used [24] for assessing the adsorption of heavy metals in soil.

### 2.3. Soil Analysis

Two different extraction methods were used to extract soil labile metals: (1) 0.01 M CaCl_2_—extractable metals and (2) 0.05 M EDTA—extractable metals. Soils from the incubation experiment were collected after 6 months of incubation, and after sample drying at 37 °C for 24 h, metal extraction was performed. Preparation of 0.05 M EDTA solution: a total of 14.61 gm of EDTA was dissolved in 300 mL of distilled water in a volumetric flask and properly mixed; after it had completely dissolved, the volume was adjusted to 1000 mL. Then, the pH was measured and adjusted to pH 7.0 with NaOH solution. The soil was extracted in a 1:5 ratio (5 g of dry soil and 25 mL of 0.05 M EDTA) and shaken for 1 h according to the modified method described by Zhang et al. [15]. Preparation of 0.01 M CaCl_2_: a total of 1.11 g of CaCl_2_ was dissolved in 500 mL of distilled water in a volumetric flask and properly mixed; after it had completely dissolved, the volume was adjusted to 1000 mL. The soil was extracted in a 1:10 ratio (2.5 g of soil and 25 mL of 0.01 M CaCl_2_) according to the method described by Houba et al. [25]. The mixture was thoroughly shaken in a rotary shaker for 2 h to achieve equilibrium and good sample dispersion. The supernatants obtained during both extraction methods and from batch sorption experiments were filtered through a membrane with a 0.45-micron pore size to remove undissolved dispersed solids and were stored at 4 °C before further analysis. The concentration of the heavy metals in the liquid supernatant was measured using a 4200 MP-AES—Microwave Plasma Atomic Emission Spectroscope (Agilent Technologies). As pH changes may influence the mobility and availability of metals, to avoid analytical errors, standard solutions (LGC Standards Ltd., Teddington, UK) for MP-AES 4200 were used for calibration, and certified reference material RTH 953 Heavy Clay Soil from LGC Promochem (LGC Standards Ltd., Teddington, UK) was used. The recovery of tested metals from Certified Reference Material (CRM) was 87–92%, and the maximum values of RSD were 3.4%. Detection limits were 0.02 mg/kg of the metal in the soil samples.

The pH of tested soil in the incubation experiment was measured twice (during and after 3 and 6 months of incubation). The soil-to-water ratio for pH measurement followed established methodologies, where 5 g of air-dried soil was equilibrated in 25 mL of deionized water for 2 h of shaking in a rotating shaker before pH determination [25], followed by the pH measurement of the supernatant with a calibrated pH-meter (Mettler Toledo, Barcelona, Spain). The pH probe was previously calibrated using standards (with known pH) to ensure the accuracy of readings.

### 2.4. Data Analysis

The experiments were carried out in duplicates. The data are presented as the mean values with relative standard deviation (RSD). Student’s *t*-tests were used to test for significant differences in pH, the metals extracted between the soils spiked with different microplastic types, and the control soil without microplastic (*p* < 0.05). The obtained data were compiled using Microsoft Excel 2021 (Microsoft, Redmond, WA, USA).

## 3. Results

### 3.1. Soil pH

**Figure 3:**
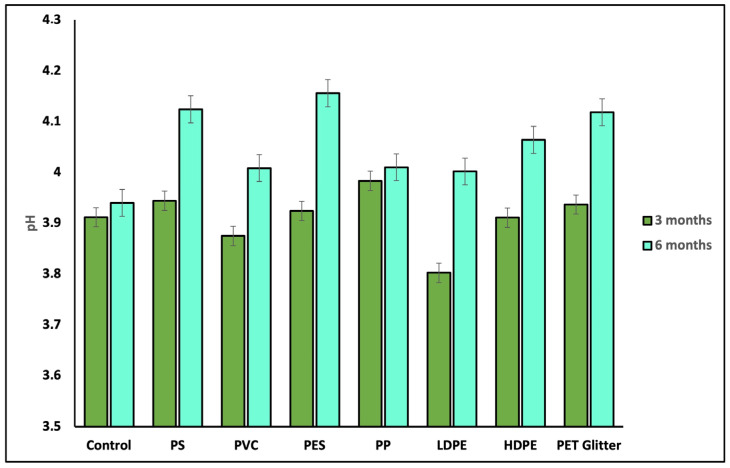
Changes in the pH of soil incubated with different microplastics. Control—without microplastic; PS—polystyrene; PVC—polyvinyl chloride; PES—polyester; PP—polypropylene, LDPE—low-density polyethylene, HDPE—high-density polyethylene, PET-Glitter—polyethylene terephthalate glitter; the results are presented as the mean average (r = 4) with standard deviation.

Soil pH increased during the incubation period in all tested microplastic variants compared to the control soil without MPs. Significantly higher values of pH were observed after 6 months of incubation. The response of the soil to the presence of microplastic particles varied between MP types. The most significant pH increase was observed in the presence of PES and HDPE particles, respectively (Figure 3). In addition to treatment with PP and PET-Glitter, pH increases between day 90 and day 180 of incubation were significantly higher (*p* < 0.05). Observed changes in soil pH between day 90 and day 180 suggest that microplastic particles have a long-term impact on soil pH, increasing soil pH significantly with time from deposition.

### 3.2. Extraction of Available Forms of Heavy Metals

**Figure 4:**
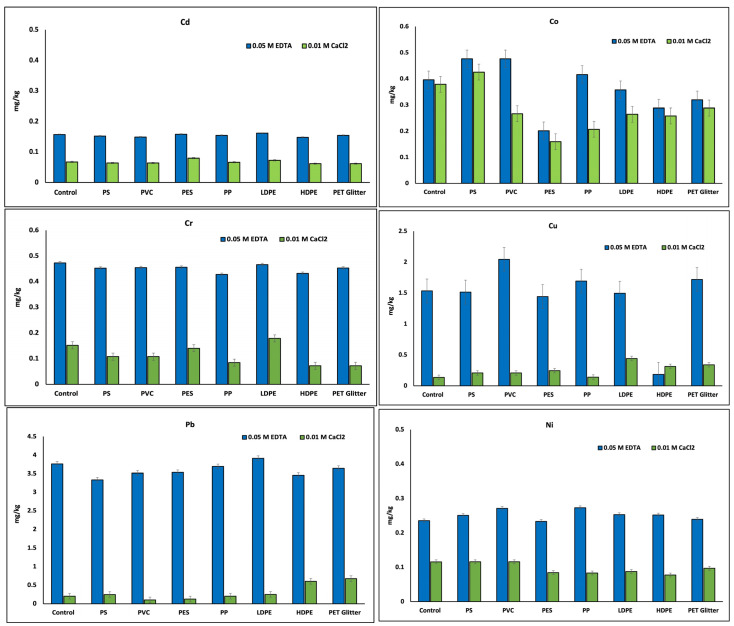
Extraction of metals with 0.01 M CaCl_2_ and 0.05 M EDTA (in mg/kg) for different combinations of heavy metals and microplastics in the soil. Control—without microplastic; PS—polystyrene; PVC—polyvinyl chloride; PES—polyester; PP—polypropylene, LDPE—low-density polyethylene, HDPE—high-density polyethylene, PET-Glitter—polyethylene terephthalate glitter; the results are presented as the mean average (r = 2) with standard deviation.

When comparing the extractants tested in the experiment, 0.05 M EDTA was much more efficient in extracting heavy metals compared to much weaker extractants such as 0.01 M CaCl_2_ (Figure 4). In terms of 0.05 M EDTA, the yields of extracted metals were similar (no significant differences) between different microplastics, and no changes due to different MP types were indicated. In contrast, for 0.01 M CaCl_2_ extraction, a tendency related to the occurrence of different MPs and changes in metal availability was observed. The most relevant changes are observed for Co, indicating that in the presence of most MPs in the soil, the bioavailability of this metal extracted by 0.01 M CaCl_2_ decreased, especially in PVC-MP, PES-MP, PP-MP, LDPE-MP, and HDPE-MP-treated soils. For PES-MP and HDPE-MP, this decrease was also indicated in the extraction using 0.05 M EDTA (Figure 2). For Cr, lower extractability using the 0.01 M CaCl_2_ extractant was observed for PS—MP, PVC-MP, PP-MP, and PET—Glitter, even though these bioavailability changes were not indicated in 0.05 M EDTA extraction. In the case of Cu, an increase in 0.01 M CaCl_2_-extracted metal was specified in the LDPE—MP, HDPE—MP, and PET—Glitter treatments, and all these changes were significant (*p* < 0.05) compared to the control soil (without MPs). For Cd, Ni, and Pb, no tendency related to MP type was indicated for both tested extractants; however, considering the trends, the amount of these three metals extracted using 0.05 M EDTA increased in the presence of microplastics in soil, yet this observation needs further investigation.

### 3.3. Sorption of Heavy Metals onto Microplastic and Soil

**Figure 5:**
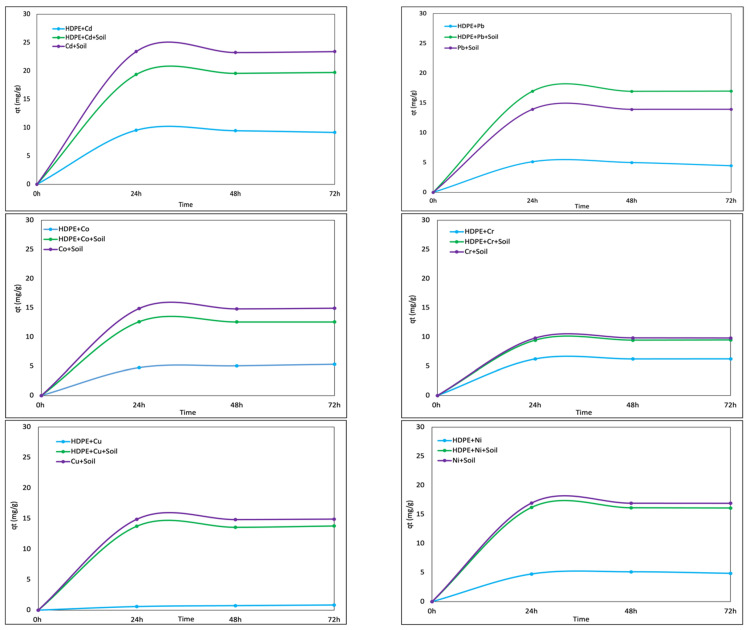
Amount of metal sorbed on different media from soil without microplastic (metal + soil).

The batch experiment showed that all tested metals were sorbed on the experimental media in different amounts, and the effect of microplastic presence in soil on the sorption of metal on soil particles also varied, depending on the tested metal. For Cd and Co, the presence of HDPE-MP decreased the sorption capacity of the soil, and more Cd and Co was sorbed on pure soil vs. soil with microplastic (Figure 3). The amount of Cd and Co sorbed on pure soil was statistically significant (*p* < 0.05). For Pb, significantly more metal was sorbed in a mix of soil with microplastic, suggesting that in the presence of microplastic, the sorption capacity of soil for Pb increased. For the other tested metals (Cr, Cu, and Ni), the change in metal sorption on pure soil vs. soil with microplastic did not indicate significant changes, and comparable amounts of Cr, Ni, and Cu were sorbed in the presence of MP particles in soil and without MP. The results of the batch experiment also showed the difference in metal sorption regarding MP, suggesting that different heavy metal cations will have different affinities to bind onto MPs. When ranking the ability of HDPE to bind the tested metals, we found the following: Cd > Cr > Co > Pb > Ni > Cu. The results of the experiment showed that the presence of MP particles increases the efficiency of Pb binding in soil, and this change is statistically significant (*p* < 0.05). Meanwhile, in terms of Cu, Cr, and Ni, there is no significant difference in metal immobilization in the presence (or not) of MPs in soil. Moreover, HDPE-MP has a very low ability to bind Cu from the solution, and this effect is also visible in a soil medium, as HDPE + Cu + Soil, Cu, and the soil treatments show no significant difference in amounts of Cu sorbed. The opposite effect was observed for Cd-treated soil, where the presence of HDPE-MP decreased the amounts of metal sorbed on the soil (Figure 5).

## 4. Discussion

Using single extraction methods for predicting the bioavailability of heavy metals (HMs) in polluted soils has always been challenging. There is no consensus about a methodology in which extractants indicate the bioavailability of HMs close to natural soil conditions with the impact of root exudates. The uptake of metals by plants is mostly facilitated by the exchangeable and water-soluble fractions of metal, which can be indicated by the use of single extraction protocols using weak acids, salts, or chelating agents like EDTA. EDTA is a powerful chelating agent that can extract a wide range of heavy metals from soil. It is highly effective in mobilizing metals by forming stable metal complexes, which can include metals bound to organic matter and other soil fractions. Studies have shown that EDTA can extract high concentrations of metals, such as Pb, Cd, Cu, and Zn, from contaminated soils and sediments [26]. For example, EDTA extracted the highest concentrations of soil-borne metals compared to other extractants like tartaric acid and water [27]. The efficiency of removing HMs from soil depends on many factors, such as the speciation of HMs in soil, the strength of EDTA, the presence of other cations in the solution, and soil pH. EDTA is a strong chelating agent that is widely used for extracting heavy metals from soil, but it does not always reflect natural soil conditions. Its high extraction efficiency mobilizes metals that might otherwise remain bound to organic matter or mineral surfaces, potentially leading to overestimated contamination risks [14]. Additionally, the persistence of EDTA in the environment can alter soil microbial activity, further affecting metal bioavailability [27]. To address these limitations, alternative extraction methods provide more ecologically relevant assessments. 0.01 M CaCl_2_ mimics natural soil solution conditions, offering a better measure of the metals available for plant uptake [22]. Low molecular weight organic acids (e.g., citric acid and acetic acid) act similarly to root exudates, influencing metal mobility in the rhizosphere while being environmentally friendly [26]. Additionally, the BCR sequential extraction method provides a more comprehensive fractionation of metals, distinguishing between exchangeable, reducible, oxidizable, and residual fractions, thereby improving remediation strategies [15]. The selection of an extraction method should depend on the study’s objective; while EDTA is useful for assessing total metal contamination, these alternatives better represent real-world bioavailability and ecological risks. The higher efficiency of EDTA extraction can be explained by its strong chelating capacity; therefore, more metals associated with organic matter can dissociate and mobilize to a solution when compared with CaCl_2_. However, the use of EDTA as a chelating agent has many opponents due to its high toxicity to soil biota and plants, chemical decomposition of organic matter, and high persistence in the soil environment [28]. The differences in extraction efficiencies between 0.05 M EDTA and 0.01 M CaCl_2_ have critical trade-offs in environmental impact, accuracy, and real-world applicability. The strong chelating properties of EDTA enable effective heavy metal extraction from soil, but its environmental persistence poses ecological risks. EDTA can enhance metal mobility beyond natural conditions, potentially increasing groundwater contamination [27]. In contrast, 0.01 M CaCl_2_ is a mild extractant that mimics soil solution chemistry, reducing environmental risks by limiting excessive metal mobilization [22]. However, its weaker extraction capacity may leave significant amounts of metals in contaminated soils, requiring additional remediation efforts. In terms of accuracy in metal bioavailability assessment, EDTA extracts both bioavailable and strongly bound metals, potentially overestimating their availability for plant uptake and leaching [14]. This can lead to misleading assessments of environmental risk and contamination severity. On the other hand, CaCl_2_ better represents the fraction of metals that are readily available to plants and micro-organisms, making it a more ecologically relevant method for assessing contamination risks [15]. Both methods have limitations in regulatory and remediation planning, affecting their real-world applicability. EDTA is useful for total contamination assessment, which is essential for determining long-term remediation needs, but it may not reflect actual exposure risks in agricultural or natural systems [26]. CaCl_2_ provides a better estimate of plant-available metals, making it suitable for agronomic and ecological risk assessments but less useful for evaluating total contamination levels that influence long-term soil health and remediation strategies [29]. Ultimately, selecting an extraction method depends on the specific environmental and regulatory context. While EDTA is valuable for total contamination assessments, CaCl_2_ is preferable for real-world bioavailability studies, particularly for sustainable soil management and ecological risk evaluation. Future studies should integrate both methods to provide a comprehensive understanding of heavy metal dynamics in contaminated soils.

The impact of microplastics (MPs) and heavy metals on soil properties and bioavailability can vary significantly across different soil types and climatic conditions. Soil texture and composition play a crucial role in determining metal mobility and MP interactions. Previous studies show that in sandy soils, lower organic matter content and larger pore spaces may lead to increased leaching of heavy metals and MPs, reducing metal retention but potentially increasing contamination in groundwater [29]. In contrast, clay-rich soils, with their high cation exchange capacity and fine particle structure, may retain more heavy metals and MPs, potentially reducing bioavailability but also prolonging contamination risks [22]. Organic matter content also influences these interactions, as soils rich in organic matter may enhance metal binding and microbial activity, affecting MP degradation and metal speciation [10]. Climatic conditions further influence these interactions. In humid regions, higher precipitation and soil moisture may enhance MP and metal transport through leaching and runoff, exacerbating environmental contamination [21]. In arid climates, lower moisture content may reduce metal mobility but promote MP fragmentation due to increased UV exposure and temperature fluctuations [7]. Temperature fluctuations in temperate regions may accelerate the aging of MPs, altering their surface properties and affecting metal adsorption/desorption dynamics [17]. Seasonal variations, including freeze-thaw cycles in cold climates, can impact soil structure and influence the stability and movement of MPs and heavy metals [30].

Finally, there is no research related to the impact of EDTA on plastic particles; however, there is a risk of plastic degradation or surface changes leading to unpredictable changes in the metal sorption/desorption process. Microplastics (MPs) significantly alter soil properties, affecting pH levels, heavy metal (HM) sorption, and bioavailability [9]. Polyester (PES) and high-density polyethylene (HDPE) increase soil pH, potentially reducing HM bioavailability [23], and MPs may also act as sinks, delaying metal release due to environmental changes [7]. MPs disrupt microbial communities and nutrient cycling, influencing soil fertility [10], and can either immobilize (e.g., Pb on HDPE) or mobilize metals (e.g., Cd and Co in HDPE-treated soils), increasing contamination risks [31].

The use of unbuffered salt solutions or low molecular organic acids should be a first choice due to their milder effects, imitating the effects of plant root system secretions and the conditions in the rhizosphere [32]. Microplastic (MP) aging significantly influences their ability to adsorb heavy metals, with various aging processes altering their surface chemistry and adsorption properties. Liu et al. [33] demonstrated that UV and high-temperature aging increase the surface roughness, crystallinity, and oxygen content of polystyrene (PS) and polylactic acid (PLA) MPs, thereby enhancing the adsorption of Cd^2+^, Cu^2+^, and Zn^2+^. Similarly, Xie et al. [34] found that different aging modes, including freeze-thaw cycles, alternating wet/dry aging, and alkali aging, increased the adsorption of Ni (II), with alkali aging exhibiting the highest adsorption and desorption rates. Zhang et al. [35] further observed that aged polyethylene (PE) and PLA MPs exhibited a 40.61% increase in Cd (II) adsorption and a 69.29% increase in Cr (VI) adsorption due to increased pore-filling, electrostatic interactions, and hydrogen bonding. The role of MP particle size in metal adsorption was highlighted by Liu et al. [33], who found that UV-aged PS MPs, particularly those at 1 µm, showed enhanced Cu (II) multilayer adsorption due to increased surface heterogeneity. Additionally, Chen et al. [36] introduced electron beam irradiation as a novel aging process that significantly increased oxygen-containing functional groups on MPs, enhancing Cr (VI) adsorption. Collectively, these studies underscore the role of MP aging in altering the environmental risks associated with heavy metal contamination, emphasizing the need for further investigation into long-term field studies and predictive modeling to assess contamination risks. Microplastics (MPs) interact with organic matter in complex ways, influencing their environmental fate and biogeochemical cycles. Chen et al. [37] found that microplastics alter the plastisphere microbiome, particularly in the presence of organic contaminants such as antibiotics, which can accelerate MP biodegradation through a priming effect, thereby affecting pollutant dynamics. Du et al. [38] investigated the interaction of algal organic matter (AOM) with MPs and observed that aging enhanced polyethylene (PE) but weakened polylactic acid (PLA) interactions with AOM, altering humification processes and surface oxidation. Wang et al. [39] demonstrated that polystyrene (PS) MPs promote the formation of refractory dissolved organic matter (DOM) while reducing CO₂ emissions by altering microbial activity, affecting carbon cycling. Liu et al. [40] reviewed the formation, characterization, and behavior of MP-derived DOM, highlighting its role in adsorption, photochemical processes, and interactions with natural DOM. Zhang et al. [41] explored MP-derived DOM interactions with iron minerals, showing that carboxyl and hydroxyl groups in MP-DOM inhibited ferrihydrite transformation, affecting mineral stability and pollutant transport. These studies emphasize the need for further research on MP–organic matter interactions, particularly in understanding their long-term ecological and biogeochemical consequences. As the uptake of metals by plants mostly originates from exchangeable forms of metals in soil [15], the number of metals extracted using CaCl_2_ better reflects the real conditions in soil solution. The findings of this study have significant broader environmental implications, particularly in agricultural systems and food safety. The presence of microplastics (MPs) in soil alters its physical and chemical properties, affecting water retention, nutrient availability, and microbial communities [10]. These changes can influence plant growth, potentially reducing crop yields and soil fertility over time [9]. Additionally, MPs can act as carriers of heavy metals, increasing the risk of metal accumulation in edible plants [16]. This bioaccumulation pathway raises concerns about food safety, as crops grown in MP-contaminated soils may contain elevated levels of toxic metals, posing health risks to consumers [11]. Moreover, MPs in agricultural lands may enter water bodies through runoff, further contributing to environmental pollution and disrupting aquatic ecosystems [42]. Understanding these interactions is crucial for developing sustainable agricultural practices and mitigating the risks associated with MPs in food production systems. The results of the study showed that metal extraction using 0.05 M EDTA was more efficient in extracting tested metals; however, the removal of most of the tested PTEs using a similar strength affected the results, and no changes and dependency regarding the presence of different MP types could be indicated in the described experiment. The use of a 0.01 M CaCl_2_ extraction seems to be more beneficial, highlighting more changes in the bioavailability of PTEs depending on the occurrence of different MP types in soil. MPs affect metal extraction efficiency, where 0.05 M EDTA extracts more metals, but 0.01 M CaCl_2_ better reflects bioavailable fractions [14], highlighting the need for refined bioavailability assessments. MPs challenge traditional remediation strategies, as they impact phytoremediation by affecting HM uptake, reduce soil washing efficiency by adsorbing chelating agents like EDTA [26], and require bioremediation strategies that integrate MP-degrading microbes [16]. Amendments like biochar can counteract MP-induced HM mobility [29].

In an aquatic environment, pH and ionic strength significantly influence the process of adsorption/desorption, as these parameters determine the reactivity and charge state of polymer surfaces [29,43]. In a more complex soil matrix, the presence of organic matter with high sorption capacity and MP surface changes due to aging can impact the process of metal sorption/desorption on soil and MPs present in the soil as contamination. The results of the incubation experiment indicated that in the presence of most of the tested MP types, soil pH increases; however, the highest pH was observed in soils treated with polyester and polyethylene microparticles. The findings of this observation support our previous study, showing that the long-term deposition of microplastic in soil increases soil pH and modifies the conditions of the metal mobilization/immobilization process [23]. An increase in soil pH leads to higher adsorption of PTEs onto soil constituents, as the precipitation of metal cations onto microplastic surfaces is enhanced [44]. Lin et al. [45] indicated that Pb (II) sorption onto MP significantly increases at pH 2.0–6.0, which can be ascribed to increasing electrostatic attraction between the negatively charged MPs and the positively charged Pb^2+^. The results of the batch experiment showed that the impact of microplastic particle presence on the sorption/desorption process of tested metals can be different; there were different improvements in soil sorption capacity for particular metals, e.g., for Pb (II), a decrease in sorption capacity in terms of Co (II) and Cd (II) and no significant impact on Cr (II), Ni (II), and Cu (II) sorption by soil in the presence of HDPE-MP. Similar findings were observed by Purwiyanto et al. [31] when comparing the sorption of Pb (II) and Cu (II) onto different microplastics (PES, PP, and PVC). In the batch experiment, the concentration of Pb on MP particles was even eight times higher compared to the amounts of Cu (II) present on the MP surface. This can be explained by the different characteristics of Pb (II) when compared to other studied metals. Pb has a higher reactivity, ion-exchange capacity, and partition coefficient, thus being more easily bound and adsorbed to microplastic. Cd (II), Co (II), Ni (II), and Pb (II) interactions with microplastics are highly dependent on pH, ionic strength, salinity, and humic acids [46]. The microplastic adsorption capacity for metal ions is also dependent on material surface properties, e.g., the presence of specific functional groups, crystallinity, a high pore volume, and surface roughness. Pristine MPs with homogeneous, smooth surfaces adsorb metal ions much slower compared with the rough surface of aged and bleached MP [47]. In general, different types of polymers achieve equilibrium for metal uptake at different times. Some studies suggest that the time for reaching equilibrium can be counted in hours but is not shorter than 100 h, while some MPs, e.g., PE, even need 8 weeks to reach their highest adsorption [48]. As simple nonpolar crystalline polymers, PE and PP have no functional groups and can only adsorb contaminants in a single layer using van der Waals forces, so the adsorption capacity is relatively small [49]. For PS and PET, the polarity is increased due to the presence of phenyl and ester groups, and the adsorption capacity can be increased [48,50]. In terms of Pb (II), the crystallinity of microplastic may be one of the essential factors influencing Pb (II) adsorption, even more important than the functional group for virgin microplastics [47]; these phenomena can explain the highest sorption of Pb (II) in the performed batch experiment with HDPE-MP. The surface properties of microplastic are also important in terms of the physical interactions between metal ions and MP. The dominant processes of cation sorption/desorption on the MP surface are attributed to van der Waals forces and π–π interactions [46]. For Cd (II) and Co (II), more ions were absorbed by pure soil, while in the presence of HDPE-MP particles, the process was disturbed, and sorption decreased. As the adsorption of Cd (II) and Co (II) occurs strictly through physical interaction, it is also strongly correlated with soil pH [51]. Previous studies confirm that PE decreases soil adsorption capacity to Cd but increases Cd desorption, thereby increasing soil Cd mobility [16,52]. The presence of MPs may increase the mobility of Cd in soil, resulting in additional health risks [53]. Much less is known about Co (II); due to being an essential trace element for many biological functions, it is not considered a pollutant. However, there are some scientific reports investigating the toxicity effects of Co (II) and microplastics in the environment. Holmes et al. [54] suggested that Co and Ni have lower affinity to be adsorbed on MP, while Rochman et al. [55] found that Pb, Zn, and Co are the metals that are adsorbed in greater quantity and that the most related polymers are PE, PET, and PP. Frost et al. [56] described the highest sorption of Pb (II), followed by Cd (II) and Cu (II), on PET microplastic fibers. Nickel is also one of the metals that are rarely studied in terms of the sorption capacity of microplastic; however, some results show that the addition of MP to sandy soil has no effect on the Ni sorption/desorption process or even that, in the presence of MP, the adsorption strength of Ni ions is reduced [57]. Tenea et al. [58] showed that Ni adsorption to microplastic is strongly correlated with polymer type, and the highest affinity for Ni adsorption can be indicated for PP; however, PS is also able to adsorb Ni ions. The results of the batch experiment showed that HDPE-MP has a low capacity for Cu and Ni sorption. This stays in agreement with previous studies by Tang et al. [30], which describe even lower Cu adsorption strength than for Ni on nylon microplastic. In the study, no significant impact of MP copresence with Cr (III), Cu (II), and Ni (II) ions in soil was indicated. The adsorption onto MPs of these metals is strongly influenced by pH. The precipitation of Cd is very likely to occur in environments with a high pH (above 7) [48], whereas, with a decrease in pH, metal ions become exchangeable and can be observed in CaCl_2_ solutions in higher amounts. The results of the study showed that the efficiency of extractions not only correlates with microplastic type but also metal type and probably the basic speciation in the soil. Most of the studies suggest that the highest extractability can be obtained for Cd (II) and Zn (II), which are weakly bound to the soil constituents and usually exist in soil in easily exchangeable and free forms in soil solutions [59]. Pueyo et al. [22] suggested that higher extractability regarding Cd and Zn can be obtained using the 0.01 M CaCl_2_ compared to a 1 M NH_4_NO_3_ solution. In another study, it was confirmed that the extractability of Cd and Cr increases with the use of chloride salts, while more Ni and Pb are extracted using chelating agents like EDTA or DTPA. Cobalt extractability in the soil is more dependent on soil properties than extractant strength or chemical characteristics [60], which also agrees with the high extractability of Co (II) using both tested agents in this study. Further implications for long-term soil health include the potential of MPs to modify soil aggregation processes, reducing water retention and increasing soil erosion risks [21]. The persistence of MPs in soil may alter microbial enzyme activity, reducing the efficiency of natural organic matter decomposition and leading to the accumulation of pollutants [61]. Additionally, MPs may serve as vectors for persistent organic pollutants (POPs), further exacerbating soil contamination and affecting remediation outcomes [42]. Strategies incorporating advanced sorbents such as engineered biochar or clay-based nanomaterials could help mitigate the retention of metals and MPs in soils [62].

Based on the research results, there is sufficient evidence to show that microplastics act as additional absorption sites for heavy metals, modifying their mobility, bioavailability, and extractability from soil. The results presented here show that the combination of microplastics and heavy metals is a key contributing factor to the sorption mechanism. Due to the complexity of the interactions between microplastics, heavy metals, and soil, more sophisticated approaches are required to develop robust sorption mechanisms for heavy metals in soil contaminated with microplastics. Future studies are therefore required to obtain more quantitative data on the relationship between microplastic and heavy metal composition in soil, as well as parameters such as pH, total carbon, and extraction efficiencies. MPs play a dual role in HM mobility and bioavailability, necessitating adjustments to current extraction and remediation techniques; this emphasizes the need for multidisciplinary research to develop sustainable soil management strategies.

These variations in extraction efficiency, which depend on metal ions, soil characteristics, or new and variable microplastic types, may require more sophisticated tools to analyze method accuracy, e.g., artificial intelligence (AI) or complex modeling of possible interactions between microplastic, metal ions, and soil characteristics. All these aspects are equally important in assessing the impact of contaminants’ presence on soil and human health.

## 5. Conclusions

This study provides critical insights into the intricate interactions between microplastics (MPs), heavy metals (HMs), and soil properties, demonstrating their significant impact on metal bioavailability, sorption processes, and soil chemistry. The findings indicate that the presence of MPs alters soil pH, with polyester (PES) and high-density polyethylene (HDPE) leading to the most pronounced pH increases. These pH changes influence metal mobilization and immobilization, affecting extraction efficiency and the overall risk associated with heavy metal contamination. The comparison of extraction methods revealed that 0.05 M EDTA was more effective in mobilizing metals from soil, whereas 0.01 M CaCl_2_ provided a more ecologically relevant measure of bioavailable metal fractions. This highlights the importance of selecting appropriate extraction techniques when assessing metal contamination risks. Additionally, the sorption experiments demonstrated that MPs serve as additional adsorption sites, with HDPE enhancing Pb immobilization while increasing the mobility of Cd and Co. These results confirm the dual role of MPs in regulating metal behavior, either facilitating immobilization or promoting mobility, depending on specific metal–polymer interactions. The implications of these findings extend beyond laboratory conditions, emphasizing the need for more sophisticated approaches to predict and mitigate the combined impact of MPs and HMs on soil health. Future research should focus on long-term MP degradation effects, interactions with soil organic matter, and environmental parameters such as ionic strength and pH fluctuations. Moreover, integrating advanced modeling techniques, including AI-driven predictive tools, could enhance our understanding of these complex interactions. Sustainable soil management strategies should incorporate remediation approaches that address MP-induced alterations in metal behavior, such as biochar amendments and microbial interventions. Overall, this study underscores the necessity for comprehensive risk assessments and innovative remediation techniques to address the growing concern of MP and HM contamination in soils. By advancing our understanding of these interactions, we can develop more effective strategies for safeguarding soil health, ensuring sustainable agricultural practices, and mitigating potential environmental risks.

## Data Availability

The original contributions presented in the study are included in the article, further inquiries can be directed to the corresponding author.

## Abbreviations

The following abbreviations are used in this manuscript:

MPs	Microplastics
HMs	Heavy metals
Pb	Lead
Cu	Copper
Co	Cobalt
Ni	Nickel
Cr	Chromium
Cd	Cadmium
PP	Polypropylene
PS	Polystyrene
PVC	Polyvinyl chloride
HDPE	High-density polyethylene
LDPE	Low-density polyethylene
PES	Polyester
PET-Glitter	Polyethylene terephthalate glitter
EDTA	Ethylenediaminetetraacetic acid
NH_4_NO_3_	Ammonium nitrate
NH_4_OAc	Ammonium acetate
CaCl_2_	Calcium chloride
POM	Polyoxymethylene
PTEs	Potentially toxic elements
DTPA	Diethylenetriaminepentaacetic acid
MP-AES	Microwave Plasma Atomic Emission Spectroscopy
RSD	Relative standard deviation
AI	Artificial intelligence
TEA	Triethylamine
pH	Potential of hydrogen
SOM	Soil organic matter
DOM	Dissolved organic matter
POPs	Persistent organic pollutants
AOM	Algal organic matter
